# Comparison of perceived quality amongst migrant and local patients using primary health care delivered by community health centres in Shenzhen, China

**DOI:** 10.1186/1471-2296-15-76

**Published:** 2014-04-29

**Authors:** Haitao Li, Roger Yat-Nork Chung, Xiaolin Wei, Jin Mou, Samuel Yeung-Shan Wong, Martin Chi-Sang Wong, Dan Zhang, Yingji Zhang, Sian Griffiths

**Affiliations:** 1 School of Public Health and Primary Care, Faculty of Medicine, The Chinese University of Hong Kong, Hong Kong, SAR, China; 2 Department of Family Practice, University of British Columbia, Vancouver, Canada; 3 Commission of Health, Population and Family Planning, Shenzhen, China

**Keywords:** Patient satisfaction, Quality of care, Primary health care, Migration

## Abstract

**Background:**

Providing good quality primary health care to all inhabitants is one of the Chinese Government’s health care objectives. However, information is scarce regarding the difference in quality of primary health care delivered to migrants and local residents respectively. This study aimed to compare patients’ perceptions of quality of primary health care between migrants and local patients, and their willingness to use and recommend primary health care to others.

**Methods:**

A cross-sectional survey was conducted. 787 patients in total were chosen from four randomly drawn Community Health Centers (CHCs) for interviews.

**Results:**

Local residents scored higher than migrants in terms of their satisfaction with types of drugs available (3.62 vs. 3.45, p = 0.035), attitude of health workers (4.41 vs. 4.14, p = 0.042) and waiting time (4.30 vs. 3.86, p < 0.001). Even though there was no significant difference in overall satisfaction between local residents and migrants (4.16 vs. 3.91, p = 0.159), migrants were more likely to utilize primary health care as the first choice for their usual health problems (94.1% vs. 87.1%, p = 0.032), while local residents were more inclined to recommend Traditional Chinese Medicine to others (65.6% vs. 56.6%, p = 0.026).

**Conclusions:**

Quality of primary health care given to migrants is less satisfactory than to local residents in terms of attitude of health workers and waiting time. Our study suggests quality of care could be improved through extending opening hours of CHCs and strengthening professional ethics education. Considering CHCs as the first choice by migrants might be due to their health insurance scheme, while locals’ recommendations for traditional Chinese medicine were possibly because of cultural differences.

## Background

China’s health challenges are similar to many other transitioning countries, where rural–urban migrants are at higher risk for many health problems than their local counterparts [1]. According to the World Health Organization (WHO), the right to the highest attainable health is a fundamental human right [2]. High quality primary health care is essential in protecting and maintaining the population’s health. China is implementing a primary health care led system through Community Health Centers (CHCs) as a strategy to create a more equitable and efficient health system. Therefore, to achieve the overall goal of better health for all, China needs to provide quality primary health care to all inhabitants including migrants. However, information on the differences in quality of primary health care delivered by CHCs to migrants and local residents, information essential for better future health care planning, is lacking.

China has a large number of internal migrants (nearly 230 million by the end of 2011) [3]. They are normally from rural areas and seeking better paid jobs in cities [4]. The term internal migrants in China usually refers to those who do not change their official *hukou* registration to the new location which they move to (i.e., floating or non-permanent residents) [5]. *Hukou* refers to a household registration status officially issued, often on a family basis, to identify a person’s official place of residence. *Hukou* defines a person’s access to employment, housing, social welfare, educational opportunities and medical and other services. Shenzhen is one of the most populous metropolitan areas located in the Pearl River Delta in Southern China, attracting millions of rural laborers to work as non-permanent migrants [4]. By the end of 2012, the number of internal migrants in Shenzhen reached nearly 12.6 million, which was about 80% of the total population of Shenzhen [6]. To facilitate migrant population management and reduce gaps between migrants and local *hukou* holders in accessing social welfare, including government-sponsored housing, education, employment and medical insurance, Shenzhen initiated its resident card system in August 2008 to replace the former, discriminatory, temporary residence regime. This new system is open to all non-hukou non-student migrants above 16 years old and has covered more than 10 million people since 2009. Registration into the system is convenient and free. A Shenzhen resident card ensures, under certain additional conditions, legitimacy for migrants to join the city’s Comprehensive Health Insurance Scheme (CHIS) as an individual (instead of as an employee within a working unit). However, due to the higher premium fee, migrants tend not to join CHIS. Whilst all employed migrants are entitled to join the Medical Insurance System for Migrant Employees (MISM), which strictly defines workers’ first contact to be with the city’s 611 primary care providers, i.e., Community Health Centers (CHCs). A referral system from CHCs exists to make sure that care in the community leads the following triage into any necessary secondary or tertiary care. Whilst CHIS participants can choose between hospital outpatient services and CHC clinics as the first contact, they generally pay higher premiums than MISM insurees.

Migrants are generally less skilled and minimally educated, and hence tend to have lower incomes than their local counterparts, leading to poorer access to health care [7]. Their lack of health insurance makes the situation even worse, resulting in unsatisfactory health outcomes [8]. Research suggests that migrants are more likely than local residents to seek health care from CHCs, the major primary health care providers in China, due to the lower costs as compared to the costs of using secondary and tertiary care facilities [9]. Given the important role played by primary health care in protecting migrants’ health, the quality of primary health care is of great concern for quality improvement for services and health improvement among migrants.

Patient satisfaction is one of the most widely used outcome indicators to measure quality of care from patients’ perspective [10]. The assessment of patients’ perception can be seen as a direct measure of quality of care received [11]. Literature indicates that quality of care, specifically patient satisfaction, is an important area because it helps physicians and health care organizations better understand patients’ points of view, and use this feedback to increase accountability and improve the services provided [12,13]. In this paper, we compare patient satisfaction with primary health care provided by the CHCs in Shenzhen between non-permanent migrants and local *hukou* residents, as well as assessing their willingness to use and recommend primary health care to others.

## Methods

### Study design, settings and data collection process

This cross-sectional study was conducted in Shenzhen in June, 2012. We recruited a site-based (i.e., CHC based) sample aged 18 years and older using a multistage random sampling process. In the first stage, 10 districts in Shenzhen were stratified into four geographical areas within or outside Shenzhen Economic Zone (SEZ), and in the eastern or western part of the city. We then randomly selected one district in each category to arrive at four districts in total, namely Luohu, Futian, Bao’an and Longgang Districts. In the second stage, we randomly selected one CHC from each randomly drawn district, so that four CHCs were chosen as study settings in total. In the third stage, we selected primary health care users through a systematic sampling method. We planned to invite 800 patients to participate in the study, 200 from each CHC. The interval for sampling was calculated by dividing the estimated total number of consultations of each CHC by the expected number of respondents to be invited in each day. The inclusion criteria for respondents were: i) aged 18 and above, ii) CHC visit at least once before the survey, iii) ability to communicate and give informed consent. Interviewers were trained extensively by the research team. Three interviewers were allocated to each CHC for five days to conduct face-to-face interviews. Patients were informed orally of the study’s purpose and then asked for their written informed consent before the interview.

### Key measures

In this study, patient satisfaction was measured by a questionnaire with 13 questions, including one question measuring overall satisfaction (Table 1). The questionnaire was based on a model by Ware et al. [14]. Factor analysis with varimax rotation was conducted to test the reliability and internal consistency of the 13 items. The Cronbach’s alpha of the scale was 0.82, indicating good reliability of the items. For consistency in response and scoring, all items were represented by a 5-point Likert-type scale (1 = very dissatisfied; 2 = dissatisfied; 3 = not sure; 4 = satisfied; 5 = very satisfied). Accordingly, a score of “1” was assigned to the lowest satisfaction rating, and “5” to the highest. Patient satisfaction score for each item was calculated by dividing the score of each item by the number of respondents for the corresponding item.

**Table 1:** Questions to measure patient satisfaction

**No.**	**Description of questions**
1	Are you satisfied with the environment of the CHC?
2	Are you satisfied with the (medical) skills of health workers?
3	Are you satisfied with the types of drugs in the CHC available to you?
4	Are you satisfied with the equipment in the CHC available for tests?
5	Are you satisfied with the charges for services?
6	Are you satisfied with the effectiveness of services (treatment outcome)?
7	Are you satisfied with the attitude of health workers?
8	Are you satisfied with waiting time (in the CHC before seeing a doctor)?
9	Is it convenient for you to come to CHC?
10	Are you satisfied with the comprehensiveness of the services?
11	Does your doctor listen to you carefully and patiently?
12	Does your doctor involve you in the decisions about your care?
13	Generally speaking, are you satisfied with primary health care?

Moreover, two questions concerning willingness to choose primary health care for usual body checks and treatment of new health problems were asked (Table 2). The willingness to recommend services, including any consultations, preventive care and Traditional Chinese Medicine, to others was estimated by three questions. The answers to these five questions were classified into two groups–“yes” (i.e., “definitely” and “probably”) and “no” (i.e., “probably not” and “definitely not”).

**Table 2:** Questions on patients’ willingness to use and recommend primary health care to others

**No.**	**Description of questions**
1	Willingness to use primary health care
	1) When you need a usual body check, do you choose primary health care as first choice?
	2) When have a new health problem, do you choose primary health care as first choice?
2	Willingness to recommend primary health care to others
	1) Would you recommend primary health care to a friend or relative for any consultations?
	2) Would you recommend Traditional Chinese Medicine to a friend or relative?
	3) Would you recommend preventive care (e.g., chronic disease management) to a friend or relative?

Socio-demographic characteristics of respondents were also collected. We grouped employment status into two groups–the respondents who had a job (including employed and self-employed) and the respondents who did not have a job (including students, housewives, the retired, and the unemployed). Information on respondents’ self-reported chronic diseases/conditions was collected. These conditions included hypertension, diabetes, cardiovascular disease, chronic respiratory or pulmonary disease, liver disease, thyroid disease, skeleton-muscular disease, gastrointestinal disorders, mental illness and disability. The respondents classified as with health insurance were those covered by any type of social health insurance scheme including MISM and CHIS. The respondents were classified into three economic groups depending on monthly household poverty line and mean monthly household income level in 2011 [15,16], i.e., below RMB 3,000/US$484 as having low income, between RMB 3,000/US$484 and RMB10,000/US$1282 as having middle income, and above RMB10,000/US$1282 as having high income. As for marital status, the respondents were classified into two groups–the singles (including never married, widowed and divorced) and the currently married.

### Statistical analysis

We compared the respondents’ socio-demographic characteristics between migrants and local residents using chi-square tests. The differences in individual and overall satisfaction mean scores between migrants and local residents were examined by both two sample *t-test* and multiple linear regressions. The differences in the willingness to use and to recommend primary health care between the migrants and local residents were tested by both chi-square tests and multiple logistic regression analyses. Confounding variables, including all characteristics of the respondents, i.e., gender, age, marital status, household income, education, occupation, health insurance status, presence of chronic diseases, health status and length of time with CHC, were adjusted in regression models. In multiple linear regression models, dummy variables were created for age, education and household income. Model fittings were conducted using backward elimination with a threshold of 0.10 for variable inclusion in the model. For all tests conducted in the study, a *p*-value less than 0.05 was considered to be statistically significant. All analyses were conducted using SPSS19.0 (IBM, USA).

### Ethical approval

Ethical approval was obtained for the study from the ethics committee of the Chinese University of Hong Kong and New Territories East Cluster Clinical Research (Ref. No. CRE-2012.441).

## Results

In total, 787 eligible respondents, of whom 140 were locals and 647 were migrants, completed the face-to-face interviews. Locals tended to be older and to have higher educational status than migrants (p < 0.001). 36.8% of locals did not have a job, while the figure was 23.2% for migrants (p = 0.003). Compared to locals, migrants were more likely to have lower income, but less likely to have any chronic existing physical, mental or psychological problems (p < 0.001). The majority of locals had health insurance (94.7%), which was higher than that of migrants (72.2%, p < 0.001). The insured migrant respondents were all covered by MISM, while the local respondents with insurance were all insured by CHIS. However, not all local residents were insured. Compared with locals, migrants had been utilizing services provided by the CHCs for a shorter period (p < 0.001). Differences found between locals and migrants in gender, marital status and health status were statistically not significant (Table 3).

**Table 3:** Socio-economic and demographic characteristics of respondents

**Characteristics**	**Locals**	**Migrants**	**P-value**
	**(n = 140)**	**(n = 647)**	
Age			<0.001
18-40	60 (42.9)	496 (76.7)	
41-59	51 (36.4)	109 (16.8)	
60+	29 (20.7)	42 (6.5)	
Gender			0.200
Female	86 (61.4)	436 (67.4)	
Male	54 (38.6)	211 (32.6)	
Marriage			0.258
Single	25 (18.2)	148 (22.9)	
Married	112 (81.8)	499 (77.1)	
Education			<0.001
Middle school and below	32 (23.0)	310 (47.9)	
High school and equivalent	46 (33.1)	212 (32.8)	
College and above	61 (43.9)	125 (19.3)	
Employment			0.003
Have a job	79 (63.2)	407 (76.8)	
Do not have a job	46 (36.8)	123 (23.2)	
Income group			<0.001
Low	11 (8.2)	111 (17.6)	
Middle	61 (45.5)	403 (63.9)	
High	62 (46.3)	117 (18.5)	
Presence of chronic diseases/conditions			<0.001
Yes	47 (34.3)	100 (15.7)	
No	90 (65.7)	536 (84.3)	
Health status			0.162
Good and above	65 (46.4)	343 (53.3)	
General and below	75 (53.6)	301 (46.7)	
Insurance group			<0.001
Has local insurance	126 (94.7)	463 (72.2)	
No local insurance	7 (5.3)	178 (27.8)	
Length of time with the CHC			<0.001
≤2 years	60 (43.8)	404 (63.2)	
>2 years	77 (56.2)	235 (36.8)	

The mean scores reported by migrants were lower than those by locals for all dimensions of satisfaction except for the environment of CHCs. Using independent two sample *t-test*, the significant differences were identified in satisfaction with types of drugs available (p ~ 0.036), effectiveness of primary health care (p < 0.001), attitude of health workers (p < 0.001), waiting time (p < 0.001), convenience (p ~ 0.013), patience of health workers (p ~ 0.001) and involvement of patients in therapeutic decision-making (p ~ 0.003). The mean score of overall satisfaction was also found to be higher for locals than for migrants (4.16 vs. 3.91, p < 0.001). However, after adjusting for the characteristics of the respondents, only the differences in the satisfaction with types of drugs available to them, attitude of health workers, and waiting time remained statistically significant (Table 4).

**Table 4:** The mean (SD) scores of different dimensions of satisfaction reported by the respondents by migrant status

**Characteristics**	**Locals**	**Migrants**	**Unadjusted P-value**	**Adjusted P-value†**
Environment	3.82 (0.798)	3.83 (0.708)	0.863	0.406
Skill of health workers	3.91 (0.800)	3.79 (0.728)	0.065	0.248
Types of drugs available	3.62 (0.901)	3.45 (0.860)	0.036	0.035
Equipment	3.54 (0.916)	3.38 (0.877)	0.051	0.104
Charges	3.96 (0.897)	3.85 (0.813)	0.148	0.754
Effectiveness	4.16 (0.736)	3.92 (0.761)	<0.001	0.098
Attitude of health workers	4.41 (0.611)	4.14 (0.769)	<0.001	0.042
Waiting time	4.30 (0.696)	3.86 (0.906)	<0.001	<0.001
Convenience	4.45 (0.579)	4.29 (0.701)	0.013	0.189
Comprehensiveness	3.86 (0.824)	3.74 (0.824)	0.107	0.561
Patience of health workers	4.26 (0.685)	4.03 (0.738)	0.001	0.370
Involvement of patients	4.23 (0.752)	4.01 (0.780)	0.003	0.457
Overall satisfaction	4.16 (0.702)	3.91 (0.691)	<0.001	0.159

†Adjusted for demographic and socio-economic characteristics of the respondents.

Compared to locals, the migrants were more likely to consider primary health care as their first choice for their usual body check (50.7% vs. 61.0%, p ~ 0.029) and usual treatment of health problems (87.1% vs. 94.1%, p ~ 0.006). The difference remained statistically significant for the latter after controlling for the characteristics of the respondents (p ~ 0.032). Locals were more inclined to recommend Traditional Chinese Medicine to others than migrants (65.6% vs. 56.6%, 0.026), after adjusting for the characteristics of the respondents (Table 5).

**Table 5:** The willingness to use by the respondents, and to recommend to others by registration type

**Indicators**	**Locals (%)**	**Migrants (%)**	**Unadjusted P-value**	**Adjusted P-value†**
Recommended to others				
For any consultations	104 (74.8)	493 (76.7)	0.660	0.457
For Traditional Chinese Medicine	82 (65.6)	305 (56.6)	0.070	0.026
For Preventive care	80 (59.3)	401 (64.1)	0.325	0.968
With primary health care as the first choice				
For body check	71 (50.7)	394 (61.0)	0.029	0.098
For usual services	121 (87.1)	609 (94.1)	0.006	0.032

†Adjusted for demographic and socio-economic characteristics of the respondents.

## Discussion

### Major findings

The findings of the study showed that the mean scores in satisfaction with types of drugs available, attitude of health workers and waiting time were higher for the locals than for migrants. Even though there was no statistically significant difference in the overall satisfaction between local residents and migrants, the latter were more likely to utilize primary health care as the first choice for their usual health problems, while the former were more inclined to recommend Traditional Chinese Medicine to their friends and relatives.

### Strengths and limitations

A particular strength of the study was that quality of primary health care was assessed from the patients’ perspective, which allowed patients to provide feedback to health care providers for quality of care improvement. The comprehensive coverage of information on health status, chronic diseases, health care measures, and socio-demographic characteristics of the respondents was another strength of this study.

There were some limitations. Firstly, the extent to which the sample was representative of the general population was limited. Only four out of more than six hundred CHCs in Shenzhen were selected for study; on the other hand, the sampling approach was CHC-based but not community-based. Even though we employed random sampling method to choose CHCs to improve the representativeness, the conclusions of the study could not be extended to the population in general. Moreover, the study was conducted in Shenzhen and the conclusion could not be generalized to other cities. Secondly, given that the scores were patient-rated, our estimates might be subject to recall bias which could not have been accounted for by statistical adjustments. However, there was no reason why the recall bias would occur systematically. Thirdly, there are other factors which could influence the quality of care provided such as language (dialect), culture and religion. These factors have not been controlled for in our regression models.

### Comparisons with existing literature

Unlike previous studies, the difference in overall satisfaction between migrants and local residents was not found in the present study after patient characteristics were controlled for. Previous studies showed a general trend toward a positive relationship between migration and poorer patient satisfaction. The study by Else et al. in Norway showed that non-western immigrants were less satisfied with visits to general practitioners than Norwegians (40.6% vs. 62.8%; p < 0.05). The study conducted in the USA by Taira et al. [17] showed that Asian-Americans rated overall satisfaction significantly lower than whites did after adjusting for potential confounders (65% vs. 72%; p < 0.01). A qualitative study in Germany which was performed on two focus groups of black immigrants from the Democratic Republic of Congo underlined that German medical staff tended to be unfriendly and to show a diffuse lack of respect towards the migrants [18]. The findings of our study differed from previous studies in that no apparent disparity was found between migrants and local residents in quality of primary health care as measured by overall patient satisfaction at multivariate analysis stage, although univariate analysis showed differences in overall satisfaction scores. The impacts that insurance and chronic diseases/conditions had on patients’ satisfaction with primary health care warrant further investigations in the future.

The mean rating score in satisfaction with the types of drugs available was higher for the locals than the migrants. In Shenzhen, two major health insurance schemes that cover out-patient services are MISM which is for migrant employees, and CHIS which mainly covers local residents. Locals who are registered with CHIS can choose health care providers without any constraint or referral from the primary health care facilities (i.e., CHCs) if secondary or tertiary care facilities were their preference for the first contact, while migrants, who are mainly covered by MISM, have to visit primary care providers and to obtain referrals from their first-contact, i.e., CHCs, to visit health facilities at higher levels (i.e., secondary and tertiary hospitals) due to health insurance reimbursement regulations. For locals, the availability at CHCs of drugs that meet their health needs, and the lower co-payment ratio at CHCs as compared with that in higher level health care facilities, may be two major reasons that motivate them also to seek health care from CHCs, even though they are given options to choose hospital out-patient services. However, since the National Essential Medicine Scheme, which aims to reduce unreasonable drug prescription and consumption, has been implemented in CHCs, the disparity found concerning drug availability was not substantial between the two groups.

The mean scores reported by the migrants for both the attitude of health workers and the waiting time were lower than those of the locals. Higher satisfaction with the attitude of health workers as perceived by the locals may reflect discrimination against migrants by the receiving societies because of their relatively lower socio-economic status. In China, the *hukou* system plays an important role in the allocation of economic resources, educational opportunities and other welfare benefits. Till now, how the *hukou* system shapes individuals’ attitudes towards disadvantaged migrants is not known. Nonetheless, Lei and Li [19] recommended that the abolition of the *hukou* system may reduce discrimination. Migrants reported to have waited longer than they expected to obtain health care services, possibly because they were mostly employed and their employment feature did not allow them paid sick leave [20]. In these circumstances they could visit the CHCs only during non-working hours, when most of their sick peers also came to visit the CHCs.

Migrants were more inclined to consider CHCs as their first point of contact, while local residents were more likely to recommend Traditional Chinese Medicine to their friends and relatives. Studies by Tung et al. [21] and Platonova et al. [22] demonstrated that patients’ satisfaction was a very strong and significant predicator of patients’ intention to choose the doctor and to recommend the primary care physician to others. Although no statistically significant difference in overall satisfaction between locals and migrants was identified in our study, significant differences in willingness to choose and to recommend primary health care were found. The choice of using CHCs as the first point of contact for migrants may be due to the structure of their health insurance scheme, which is often interrelated with their employers and working contracts, but may not necessarily be related to the quality of the services per se [9,23]. Traditional Chinese Medicine services, which were provided by CHCs as signature services, might be another reason prompting locals who were older as a group to seek health services from CHCs and to recommend them to others. This might reflect cultural belief differences between the old and the young [24].

### Implications for research and practice

Local residents and migrants in Shenzhen appeared to be equally satisfied with the quality of primary health care as measured by the overall patient satisfaction score. While this might reflect the true situation, we need to be aware that the respondents in this study were restricted to CHC users and caution should be used when interpreting the findings. Future studies using other indicators measuring primary health care quality, such as primary health care experiences as well as studies amongst non-CHC users, should be conducted to ensure diversity in the subjects.

The findings showed that migrants were reliant on the primary health care system, especially western medical services delivered by the CHCs, more so than the locals. In case disparity in actual waiting time (as opposed to perceived waiting time) really existed between migrants and locals, it would be a greater concern to the migrants who solely relied on the services under insurance constraint but were treated differently at CHCs, as compared to the locals, who would have more options in seeking care. Extending opening hours of CHCs may be one possible solution from the health care providers’ side to reduce migrants’ visits during peak hours, and thus shorten waiting times In addition, policies to encourage employers to provide paid sick leave to employees for health care should be considered. Furthermore, although the abolition of the *hukou* system may be an alternative to reduce discrimination (e.g., attitude) by health workers against migrants in the future, how to strengthen professional ethics education for health workers in CHCs should be considered by policy makers.

## Conclusions

In summary, quality of primary health care given to migrants is less satisfactory than to local residents in terms of the attitude of health workers and waiting time. Our study suggests that extending opening hours of CHCs and providing paid sick leave for health care are possible approaches to shorten waiting time for migrants, while abolishing the *hukou* system and strengthening professional ethics education should be considered to reduce discrimination. Considering CHCs as the first choice by migrants might be due to their health insurance scheme, while locals’ recommendations for TCM were possibly because of cultural differences.

## Competing interests

The authors declare that they have no competing interests.

## Authors’ contribution

XLW, SG, SW and MW conceived of the study, and took part in its design. DZ and YJZ participated in the data collection and helped to draft the manuscript. HTL, RC and XLW were responsible for data analysis and interpretation. HTL, RC and XLW drafted the manuscript. XLW, SW, MW, JM and SG revised the draft for intellectual content. All authors read and approved the final manuscript.

## Pre-publication history

The pre-publication history for this paper can be accessed here:

http://www.biomedcentral.com/1471-2296/15/76/prepub
